# Application of the ORBEYE Three-Dimensional Exoscope for Parotidectomies

**DOI:** 10.3390/jcm14010047

**Published:** 2024-12-26

**Authors:** Masao Yagi, Tomofumi Sakagami, Minaki Shimizu, Yuhei Ogino, Mizuki Morita, Hiroto Kawasaki, Atsushi Tomoda, Yasutaka Yun, Kensuke Suzuki, Takuo Fujisawa, Hiroshi Iwai

**Affiliations:** Department of Otolaryngology, Head and Neck Surgery, Kansai Medical University, Shinmachi 2-5-1, Hirakata 573-1010, Osaka, Japan; sakegami1981@yahoo.co.jp (T.S.); minaki1226@yahoo.co.jp (M.S.); anv86728@yahoo.co.jp (Y.O.); moritmiz@hirakata.kmu.ac.jp (M.M.); ernest.t.s.1230@gmail.com (H.K.); pegu04@gmail.com (A.T.); yunys@hirakata.kmu.ac.jp (Y.Y.); suzukken@hirakata.kmu.ac.jp (K.S.); fujisawt@hirakata.kmu.ac.jp (T.F.); iwai@hirakata.kmu.ac.jp (H.I.)

**Keywords:** three-dimensional, parotid tumor, exoscope, facial palsy, parotidectomy

## Abstract

**Background/Objectives:** Parotid surgery is generally performed with the naked eye or using surgical loupes. However, this approach has technical disadvantages. Therefore, this study aimed to compare the use of an exoscope with that of loupe for parotidectomies. **Methods:** A retrospective review of patients who underwent partial parotidectomies for parotid tumors was conducted. We examined the approach (anterograde/retrograde), tumor localization (superficial/deep), operative time, volume of intraoperative blood loss, and postoperative complications occurring within 6 months. **Results:** Overall, 174 patients underwent parotidectomies (90 in the exoscope group, 84 in the loupe group). In parotidectomies using the anterograde approach, the exoscope group had significantly fewer reports of facial nerve palsy than the loupe group. Parotidectomy-related complications other than facial palsy were significantly fewer in the exoscope group. However, the operation time was significantly longer in the exoscope group than in the loupe group. **Conclusions:** An ORBEYE^TM^ exoscope provides a magnified view of the surgical field, leading to more accurate operations in parotid surgery and potentially fewer complications.

## 1. Introduction

One of the most challenging aspects during parotid surgery is to properly resect the tumor while preserving the facial nerve. The key aspect in achieving this lies in being able to locate the tumor while clearly visualizing the nerve. Surgical interventions on small anatomical structures such as the facial nerve and Stensen’s duct require magnification of anatomical details to perform parotid surgery with precision. Surgical loupes and microscopes have been conventionally used to view nerves and ducts under magnification. However, both have disadvantages such as a narrow field of view and interference of equipment; therefore, using these instruments compromises posture or create positional discomfort occasionally.

Recently, three-dimensional (3D) exoscope technology has been introduced in the clinical practice and is becoming popular among several surgical specialties, such as neurosurgery, reconstructive microsurgery, and otologic surgery [1,2,3]. In these fields, the exoscope is primarily used as an alternative to a microscope. An exoscope allows the surgeon to visualize the surgical field from various angles without looking through the eyepieces of a microscope while reducing physical stress as the procedure is performed through a monitor. Several studies, including neurosurgery, microsurgery, and otology, have demonstrated that the use of exoscopes results in superior ergonomics, which can reduce surgeon fatigue caused by fixed unnatural posture [1,2,3]. The main disadvantage of an exoscope is its limited depth perception, which has been overcome by using 3D-4K monitors [4]. Only a few reports exist on the utility of 3D exoscopes in parotid surgery [5,6,7]. Furthermore, reports on whether this technology is beneficial in parotid surgery or if it offers advantages over conventional techniques is lacking.

Several exoscope products are available in the market, and they all display high-resolution images on a flat display with different types of mirror bodies. In head and neck surgery, the focal distance is close and a large mirror body would interfere with surgical operations. The ORBEYE^TM^ 4K-3D exoscope (Olympus, Tokyo, Japan) is FDA-approved, features a variable 12× optical zoom, automatic calibration, and focuses with easy adjustment, aspiring to offer optimal visual fidelity. The ORBEYE^TM^ exoscope with its small mirror body and excellent autofocus function is a suitable tool that can be used in head and neck surgery to secure the operative field and transfer the field of view without stress.

The position of the exoscope when being used as an alternative tool to the surgical microscope in neurosurgery differs from that being utilized during parotid surgery. Generally, microscopes are not used and surgery is mainly performed with the naked eye or surgical loupes in parotid surgery. Therefore, this study aimed to examine the utility of the ORBEYE^TM^ exoscope for parotidectomies since it may contribute not only to improving the accuracy of surgery but could also be an alternative tool. Furthermore, we compared the two techniques (exoscope vs. loupe) to assess the differences in operation time, volume of blood loss, and complications related to parotidectomies.

## 2. Materials and Methods

### 2.1. Ethics

The study was approved by the Institutional Review Board at the Kansai Medical University (Approval No. 2020076). The hospital Ethics Committee exempted this study from the need to obtain consent from the patients.

### 2.2. Participants

This retrospective study included patients who presented with parotid tumors and underwent a partial parotidectomy at the Kansai Medical University—Otolaryngology, Head and Neck Surgery Department between January 2018 and June 2023. A retrospective review of all medical records of these patients clinically diagnosed with parotid tumors was performed.

The study population was divided into two groups based on the use of an exoscope: the loupe group (before March 2021, the date of exoscope introduction at the hospital) and the exoscope group (after March 2021 to June 2023).

Patients diagnosed with a benign or low-grade malignant parotid tumor qualified for a partial parotidectomy. A partial parotidectomy included any procedure in which the facial nerve trunk or branches of the facial nerve were exposed but only a part of the superficial or deep lobe was removed.

The exclusion criteria of this study included (i) simultaneous neck dissection, (ii) a high-grade malignancy that required a total parotidectomy, (iii) the tumor extending into the parapharyngeal space, (iv) previous parotid surgery exposing the facial nerve, and (v) the resection of more than four tumors.

### 2.3. Parotidectomy

Parotidectomies were performed as follows: modified Blair incision; flap creation; identification of the great auricular nerve; and identification of facial nerve in the anterograde or retrograde approach. The retrograde approach was guided by either the marginal mandibular branch, buccal branch, or zygomatic branch. As described in the indication criteria for parotidectomies in the retrograde approach reported previously [8], the indication for the retrograde approach is the upper edge of the tumor below the mastoid tip, and for tumors located higher up, the indication is a mass of ≤2 cm anterior to the posterior border of the mandible.

The surgeon was experienced in head and neck surgery for more than 20 years and had performed more than 200 parotidectomies. Electromyographic monitoring with NIM-Response 3.0 (Medtronic Japan, Tokyo, Japan) was used in all the operations. The surgeon performed the parotidectomy with either ×2.5 magnifying loupes or the ORBEYE^TM^ exoscope, with no changes being made during the procedure. We retrospectively examined the approach (anterograde/retrograde), tumor localization (superficial/deep), duration of surgery, volume of intraoperative blood loss (deduced by a combination of gauze weighing and measuring the contents after deducting the saline from the total volume of the suction bottle), postoperative facial nerve palsy, and other complications, such as postoperative hemorrhage, infection, sialocele, and Frey’s syndrome occurring within 6 months of the operation. On the first postoperative day and several days postoperatively, the facial nerve function and other complications listed were evaluated by a different surgeon than the one who performed the surgery. The analyzed cases were followed up for at least 6 months. In the loupe group, the operation times for the first year and a half were compared to that of the second year and a half. In the exoscope group, the operative time was compared between the first 1 year and 2 months and for the second 1 year and 2 months.

### 2.4. Statistical Analysis

A comparative analysis was performed using JMP^®^ version 16 (SAS Institute, Cary, NC, USA) for Windows 10.0 statistical software. A Fisher’s exact test was used for sex–gender, location, anterograde and retrograde differences, and complications between the groups; a Wilcoxon signed-rank test was used for age, operative time, and blood loss. Statistical significance was set at *p*-values less than or equal to 0.05.

## 3. Results

During the study period, 174 patients underwent parotidectomies using either the loupes (loupe group: n = 84) or ORBEYE^TM^ exoscope (Exoscope group: n = 90). All parotidectomies were completed uneventfully, without any intraoperative complications. In the exoscope group, an ORBEYE^TM^ was used throughout the surgery and was not taken off during the operation.

The mean age of the loupe group was 55.25 (range, 12–83) years (SD 16.44), compared with 55.33 (range, 12–81) years (SD 17.13) in the exoscope group (*p* = 0.9916). Patient characteristics are summarized in Table 1. No significant difference was found between superficial and deep lobe tumors. The loupe group had slightly more parotidectomies using the retrograde approach than the exoscope group; however, the difference was not significant. The most common histologic types in the loupe vs. exoscope groups were pleomorphic adenomas (41 vs. 45) and malignant cases (9 vs. 4), respectively, indicating that the distribution of histologic types was relatively equivalent. The loupe group had 7 T1 and 2 T2 malignancies, while the ORBEYE group had 2 T1 and 2 T2 malignancies. The size of malignant tumors in the two groups ranged from 7 to 22.5 mm (mean 16.32 mm) and 10 to 32 mm (20.65 mm), respectively, with no significant between-group differences. Operative time comparisons between the malignant cases in the two groups resulted in no significant differences (loupe group: 78–178 min [mean 122.44 min]; ORBEYE group: 106–166 min [mean 133 min]). Blood loss comparisons for the malignancies in the two groups were also not significantly different (loupe group: 5–60 mL [mean 20.66 mL]; ORBEYE group: 29–50 mL [mean 33.5 mL]). There were no facial nerve invasions, postoperative facial nerve palsy, or other complications among the malignant tumor cases in either group.

**Figure 1:**
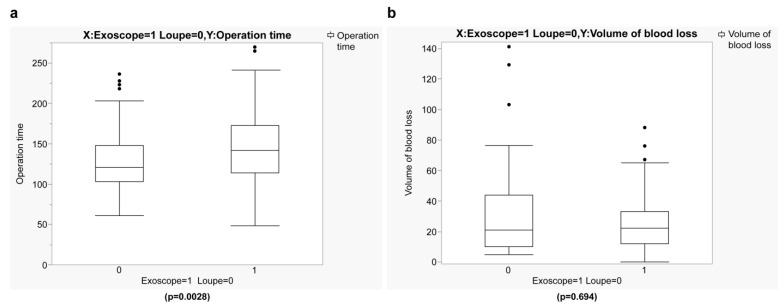
Comparison of operative time between the exoscope and loupe groups. (**a**) Operative time (*p* = 0.0028) and (**b**) volume of blood loss (*p* = 0.694).

Surgical variables and outcomes are shown in Table 2 and Table 3. The exoscope group’s mean operative time (145.8 min) was significantly longer than that of the loupe group (128.5 min) (*p* = 0.0028) (Figure 1a). The mean amount of blood loss in the exoscope group (25.2 mL) was lower than that of the loupe group 29.3 mL); however, this difference was not significant (*p* = 0.694) (Figure 1b).

Complications, including facial paralysis, are shown in Table 2 and Table 4. No permanent facial palsy was observed in either group. Postoperative transient facial nerve palsy was more common in the loupe group (5/84 patients; 5.95%) than that in the exoscope group (1/90 patients; 1.11%), although there are no significant differences between the procedures. In the retrograde approach, there was only one case of facial paralysis in each group and there was no difference between the two groups; however, the exoscope group had significantly fewer cases of facial palsy than that in the loupe group in the anterograde approach (Table 4). Complications other than facial nerve palsy related to parotidectomies were significantly less common in the exoscope group, with three cases (3.33%) being seen in the exoscope group compared to 10 cases (11.11%) in the loupe group (Table 3).

**Figure 2:**
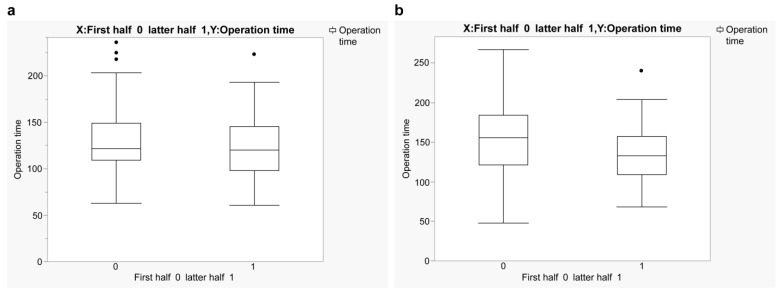
Comparative results on surgical time of the first and latter halves in the exoscope and loupe groups. (**a**) Loupe group (*p* = 0.700) and (**b**) exoscope group (*p* = 0.007).

The loupe group showed no significant difference in operative time between the first and latter halves (Figure 2b). In contrast, the exoscope group showed a significant reduction in operative time between the first (mean: 156.5 min, SD: 48.3) and latter (mean: 137.1 min, SD: 37.6) halves (Figure 2a).

## 4. Discussion

We conducted a retrospective analysis of 174 patients undergoing a partial parotidectomy at our department with the loupe or exoscope. Our findings revealed that, in parotidectomies using the anterograde approach, the exoscope group had significantly fewer facial nerve palsies than the loupe group. In addition, parotidectomy-related complications other than facial palsy were significantly fewer in the exoscope group. Most of the variables indicated better outcomes in the exoscope group compared with those in the loupe group. However, the operation time was significantly longer in the exoscope group, which differed by approximately 17 min on average. Notably, operation time was significantly reduced the following year compared with that in the first year of the exoscope use.

The exoscope has several advantages over existing instruments (operation microscopes, endoscopes, and loupes), including high-resolution 3D visualization, improved ergonomics with reduced fatigue, a higher degree of freedom of movement, and valuable educational aspects [2,3,9,10]. Of these advantages, one of the most significant benefits of this technology is the ability to project high-definition images on an external monitor, allowing the entire surgical team to share the same viewpoint as that of the surgeon. This provides all staff, especially residents in training, the opportunity to learn essential skills for parotid surgery. Comparatively, this is not possible with the use of loupes. The physical advantages of the ORBEYE^TM^ in parotid surgery are that the small mirror body minimizes interference in the surgical field, and excellent autofocus function allows the surgeon to maintain a good surgical field of view without stress. Bulky operative microscope heads interfere with surgical instruments and hands during head and neck surgery and sometimes need to be removed from the operative field, while smaller exoscope heads can be placed away from the surgical field, allowing for the use of surgical instruments and hands without their removal. Although parotid surgery using the naked eye or a loupe does not cause instrumental interference, the field of view of the loupe is much narrower than that of the exoscope and the field resolution of the loupe is considerably inferior to that of the exoscope.

Significantly longer operative times were observed in the exoscope group compared to the loupe group. The use of an exoscope has increased the operative time in several field surgeries [2,9]. Compared with the use of loupes, which is similar to visual observation, parotid surgery performed with a magnified field of view while looking at a monitor requires hand-eye coordination, i.e., more careful and gradual surgical movements, which may have contributed to the longer operative times. Additionally, depth perception in parotidectomies using an exoscope might be inferior to that provided by loupes and microscopes, as described earlier [5].

As seen with the introduction of many new technologies, a learning curve is associated with the use of the exoscope [11]. However, exoscopes have a shorter learning curve than that of the operating microscope [11,12]. In this study, a significant operative time reduction of approximately 10 min in the exoscope group was observed. This suggests that the difference in the operative time of exoscopic parotidectomes may be reduced by the learning curve.

The facial palsy rate was 6.0% in the loupe group and 1.1% in the exoscope group, relatively lower than that reported in the literature [13]. A systematic review of facial nerve monitoring during parotidectomies indicated 22.5% of facial nerve palsy under facial nerve monitoring [13]. A prospective, randomized single-center study of a parotidectomies with a 3D exoscope using VITOM 3D (Karl Storz, Tuttlingen, Germany) demonstrated a valid visualization, but a higher rate of transient nerve palsy was seen, and five patients with deep lobe parotidectomies required intraoperative conversion with a microscope [5]. In the present study, switching to loupes or conventional microscopes was not necessary when operating on deep lobe tumors. The fact that we completed deep lobe tumor surgery successfully with an ORBEYE^TM^ exoscope may be attributed to the ease of operation of ORBEYE^TM^ compared with other exoscopes, especially since only one case with postoperative facial palsy was observed in the exoscope group. Using the ORBEYE^TM^ exoscope may improve the rate of facial nerve palsy because it allows for detailed visualization of the facial nerve anatomy due to its increased degree of freedom for adjustment. More detailed visualization of the facial nerve and surrounding tissues by the ORBEYE exoscope may improve the precision of surgical manipulation, thus reducing the stress on the facial nerve. Malignant tumors were analyzed in both groups, and only tumors smaller than T2 (the mean sizes of the tumors were 16.32 mm [Loupe] and 20.65 mm [ORBEYE] along their largest diameters) were included. None of the malignant tumors exhibited facial nerve invasions, postoperative facial nerve dysfunction, or other complications. Moreover, no between-group differences were revealed for malignant tumors in operative time and blood loss. Therefore, it is unlikely that the greater number of malignant tumors in the loupe group could have influenced the comparison of facial nerve palsy rates and other complication rates between the two groups. Retrograde nerve dissection is less likely to cause facial paralysis owing to the rich network of facial nerves; only one case of paralysis was reported in each group. Although some may believe that the lack of permanent paralysis in the two groups makes no difference to the long-term impact on the patient, the physical and psychological impact of even transient paralysis on the patient in the short term cannot be ignored, as recovery from postoperative facial paralysis often takes several months.

The levels of complications other than facial nerve palsy in the exoscope group were significantly lower compared with those in the loupe group. In the systematic review of minor parotidectomy complications, hematoma, wound infection, and sialocele were at 2.9, 2.3, and 4.5%, respectively [14]. However, in the present study, hematoma, wound infection, and sialocele were all at 1.11% in the exoscope group and at 1.19, 1.19, and 7.14%, respectively, in the loupe group. The use of an exoscope tends to reduce minor complications because it is easier to visualize minute blood vessels and the Stensen’s duct in a magnified view, which is usually difficult during resection with the naked eye or using loupes. Further studies are necessary to determine whether complications are indeed reduced by using the exoscope. Future prospective and randomized studies regarding exoscopic parotidectomy are warranted.

Risk factors for complications, including facial paralysis, have been previously reported, including tumor grade, extent of resection, age, tumor size, cases of reoperation, operation time, and deep lobe tumors [15]. The bias arising from different tumor locations, sizes, and extent of resection between the groups remains a limitation in this study. However, all cases in the two study groups were partial parotidectomy cases, suggesting that there is not a strong difference in the extent of resection. In addition, the reoperation cases were omitted, although the operation time was longer in the exoscope group. Furthermore, the number of superficial and deep lobe tumors was not significantly different between the two groups. Hence, there appeared to be no significant difference in the risk factors for complications between the two groups in this study.

The initial startup cost of a 3D exoscope ranges between USD 250,000 and USD 1,500,000, and the cost of ORBEYE^TM^ is USD 412,000, which is similar to that of a conventional state-of-the-art microscope with similar configurations [16]. The only consumables include disposable sterile drapes, which cost USD 56 each. Endoscopic and robotic parotid surgeries have the same advantages as an exoscopic parotidectomy in terms of magnification, and patients with strong cosmetic needs benefit from them [17]. However, it is difficult to fully expose all surgical fields simultaneously under endoscopy and robotic surgery. In addition, in parotid tumor resection, since the location and margin of the tumor can be confirmed only through palpation, we believe that an exoscopic parotidectomy is more useful than the other two in that it allows for direct palpation. Further studies are needed, including comparison with devices and cost-effectiveness investigations, with endoscopic, robotic, and exoscopic parotidectomies to reveal their real potential in clinical practice.

Some surgeons may be reluctant to perform parotid surgery using the ORBEYE^TM^, especially those who operate with the naked eye, as they may face hurdles while using a monitor. However, as mentioned earlier, there are minor issues regarding interference with the mirror body which can be overcome with experience when using the ORBEYE^TM^. With experience, the benefits of using magnification to better visualize the facial nerve and Stensen’s duct could be felt. The ability to observe such fine structures accurately may extend the surgeon’s career by reducing surgeon fatigue caused by maintaining fixed and unnatural stances. The main limitation of this study is that it was a retrospective study based on one surgeon’s experience. Therefore, future prospective studies of parotid surgery should be conducted to compare the conventional method with the ORBEYE exoscope approach in a larger number of patients being seen by multiple surgeons.

## 5. Conclusions

We evaluated the use of the ORBEYE^TM^ exoscope for parotidectomies in comparison with using a loupe. In parotidectomies using the anterograde approach, the exoscope group had significantly fewer experiences with facial nerve palsies than the loupe group. Although parotidectomies using an exoscope increased the operative time, the complication rate other than facial palsy was significantly decreased, and most of the variables, except operation time, yielded better results using the exoscope, suggesting that it is highly useful in parotid surgery. Currently, parotid surgery is generally performed with the naked eye or surgical loupes. We believe that the ORBEYE^TM^ exoscope is not just an alternative tool but also much more beneficial than loupes owing to its improved accuracy and performance in parotid surgery.

## Figures and Tables

**Table 1:** Demographic and clinical characteristics of patients.

	Loupe Group (n = 84)	Exoscope Group (n = 90)	*p*-Value
Sex, M/F	36/48	41/49	0.7613
Age (y)	55.25 (12–83)	55.33 (12–81)	0.9916
Histology			
Pleomorphic adenoma	41	45	
Warthin’s tumor	20	23	
Other benign tumors	14	18	
Malignant tumor	9	4	
Location of deep/superficial tumors	15/69	11/79	0.4017
Approach—anterograde/retrograde	38/46	48/42	0.2197

**Table 2:** Comparative results on postoperative facial nerve palsy between the two procedures (loupe vs. exoscope).

Facial Nerve Palsy	Loupe Group (n = 84)	Exoscope Group (n = 90)	*p*-Value
Total	84	90	
None	79 (94.05%)	89 (98.89%)	
Temporary	5 (5.95%)	1 (1.11%)	0.1078
Permanent	0	0	
Anterograde approach	38	47	
None	34 (89.47%)	48 (100%)	
Temporary	4 (10.53%)	0 (0%)	0.0348 *
Retrograde approach	46	42	
None	45 (97.83%)	41 (97.62)	
Temporary	1 (2.17%)	1 (2.38%)	1.0000

* statistical significant.

**Table 3:** Comparative results on postoperative complications other than facial nerve palsy between the two procedures (loupe vs. exoscope).

Complications	Loupe Group (n = 84)	Exoscope Group (n = 90)	*p*-Value
Other complications—total	10 (11.90%)	3 (3.33%)	0.0426 *
Sialocele	6 (7.14%)	1 (1.11%)	0.0573
Postoperative bleeding	1 (1.19%)	1 (1.11%)	1.0000
Wound infection	1 (1.19%)	0	1.0000
Frey syndrome	1 (1.19%)	0	1.0000
First bite syndrome	1 (1.19%)	1 (1.11%)	1.0000

* statistical significant.

**Table 4:** Comparative results on surgical variables between the two procedures (loupe vs. exoscope).

Surgical Variables	Loupe Group (n = 84)	Exoscope Group (n = 90)	*p*-Value
Operation time			
All procedures	128.5 (n = 84)	145.8 (n = 90)	0.0045 **
Anterograde	155.7 (n = 38)	166.8 (n = 48)	0.1654
Retrograde	106.0 (n = 46)	121.8 (n = 42)	0.0078 **
Volume of blood loss			
All procedures	29.3 (n = 84)	25.2 (n = 90)	0.6212
Anterograde	39.6 (n = 38)	29.5 (n = 48)	0.2655
Retrograde	20.8 (n = 46)	20.3 (n = 42)	0.7201

** statistical significant.

## Data Availability

The data that support the findings of this study are available from the corresponding author, Masasao Yagi, upon reasonable request.
